# Comparison of Single Session Auditory Versus Visual Feedback on Performance and Postural Balance in Hemiplegic Children With Cerebral Palsy

**DOI:** 10.7759/cureus.64003

**Published:** 2024-07-07

**Authors:** Ghaith Fadhil Lafta Alhashimi, Azadeh Shadmehr, Sara Fereydounnia, Behrouz Attarbashi Moghadam, Firas Mohammed Abdulgani

**Affiliations:** 1 Physical Therapy, School of Rehabilitation, Tehran University of Medical Sciences, Tehran, IRN; 2 Orthopaedic Surgery, School of Medicine, Al Nahrain University, Baghdad, IRQ

**Keywords:** children with disabilities, postural balance, biofeedback, spastic hemiplegia, cerebral palsy

## Abstract

Background: Cerebral palsy (CP) is a pediatric disorder characterized by a motor impairment resulting from a permanent, non-progressive lesion in the brain. Cerebral palsy is marked by movement and postural control impairments, which greatly affect body structure, function, daily activities, and participation.

Objective: To compare the single-session auditory versus visual feedback on performance and postural balance in children with hemiplegic cerebral palsy.

Method: The study was a crossover clinical trial involving a group of 25 patients diagnosed with CP hemiplegia, aged between 6 and 12 years, including both genders. Each patient underwent conventional balance therapy followed by either auditory feedback or visual feedback intervention. After a 48-hour wash-out period, they received conventional balance therapy again before undergoing the alternative intervention initially assigned. The Modified Ashworth scale (MAS), pediatric balance scale (PBS), timed one-leg stance, time up and go test (TUG), and center of pressure (CoP) displacements were assessed as the outcome measures before and after the interventions.

Results: Based on the one-leg stand test, TUG, and CoP displacement outcome measures results, both interventions improved balance time, speed of movement, and postural stability in children with hemiplegic spastic cerebral palsy (P < 0.05). Moreover, after a single session of the intervention, the visual feedback group demonstrated a significantly greater improvement in the TUG test, one-leg stand test, and CoP displacement compared to the auditory group (P < 0.05).

Conclusions: The results of the study suggest that combining auditory or visual feedback with conventional balance therapy is effective in treating children with hemiplegic spastic cerebral palsy; furthermore, the utilization of visual feedback would be more effective. Further research is needed to determine the long-term effects of visual and auditory feedback on the assessed outcome measures.

## Introduction

Cerebral palsy (CP) is the leading cause of childhood physical disabilities, affecting around 2 to 3 children per 1,000 live births [1-3]. CP is caused by damage to one or more areas of the developing brain, which affects body movements, posture, and coordination [4].

In Australia, the most frequent form is hemiplegic CP (HCP), where one body side is affected as a result of brain damage that affects one hemisphere [5]. In Iraq, 43% of the cases were spastic diplegic CP, 19% were hemiplegic CP (HCP), 15% were quadriplegic CP, 7% were athetoid CP, and 16% were other or unclear (mixed or ataxic CP) [6]. Symptoms of HCP range widely from spasticity, impaired motor organization and functioning, movement impairments, lack of postural control, changes in walk patterns, balance problems, trunk, and lower extremity motor control impairments to cognitive and intellectual problems [7]. Furthermore, bilateral and hemiplegic spasticity give rise to difficulties in posture, balance, and gait control [8-10].

Spasticity manifests as an increased stretch reflex, which is intensified by movement at high velocity. The lack of modulation of the stretch reflex causes premature and/or exaggerated muscle contraction that may resist the passive stretch. Therefore, spasticity can directly impact motion control and maintenance of postural balance [11]. The loss of balance ability is a clinically important problem for children with cerebral palsy, and it becomes an important factor in improving quality of life through daily living activities [12]. Balance in children with CP is impaired by the poor postural control mechanism; approximately 33% of the children diagnosed with CP exhibited abnormal values in a center of pressure measure [13]. Earlier studies on balance have found that children with CP had poorer static and dynamic balance reactions than those of typically developing children [13,14]. These balance problems increased the risk of falls, which further affected children with CP in their performance of activities of daily living (ADL), mobility, and participation [15].

Biofeedback methods have been utilized within the realm of physical therapy for over half a century, proving to be advantageous in the treatment of neuromuscular conditions. The incorporation of biofeedback techniques, both auditory and visual, has demonstrated positive outcomes when integrated into physical therapy regimens for patients with motor impairments or dysfunction following a stroke, orthopedic surgery, or as a result of other neuromuscular ailments [16]. 

Previous studies mainly focused on interventions that improve the balance ability of children with cerebral palsy. They applied neurodevelopmental therapy (NDT), hippotherapy, treadmill training without weight bearing, body trunk muscle strengthening training, reactive balance training using a moving platform, visual feedback training, auditory feedback training, and others. A study was conducted to assess the impact of visual and auditory cues on gait training for patients with cerebral palsy-related gait disorders. The results indicated that training with visual and auditory feedback cues can lead to improvements in the walking abilities of these patients [17]. Additionally, a study focused on finding a more effective balance training method for children with cerebral palsy. It revealed that visual and auditory feedback was likely effective in improving standing balance in these children [18]. Furthermore, a comparison was made between the effects of visual and auditory biofeedback during sit-to-stand training on 35 chronic stroke patients. The findings suggest that sit-to-stand training with visual and auditory feedback may enhance both sit-to-stand performance and balance ability in stroke patients [19].

Hence, the purpose of this study is to examine the difference between balance training along with visual feedback and balance training using auditory feedback on balance improvement in children with HCP.

## Materials and methods

Study design

The study was a crossover randomized clinical trial involving single-blinded targeted children with hemiplegic spastic cerebral palsy (HSCP) of both genders. These children were referred by a neurologist and met specific inclusion criteria: age between 6 and 12 years, grade 1 or 2 on the Modified Ashworth scale for ankle plantar flexors and quadriceps muscles, Gross Motor Function Classification System levels I or II, absence of uncorrected vision or hearing impairments, ability to follow verbal instructions, and ability to stand without lower limb braces. Exclusion criteria included other neurological conditions, leg length or muscle shortening, pes cavus, recent fractures, conditions threatening stability, and vestibular or optical pathology. The study included 25 children with HSCP who received conventional balance therapy, then auditory or visual feedback interventions after a 48-hour washout time period were used. children received conventional balance therapy, then the opposite intervention that was received first. This study design was used to maintain consistency in age and affected side distribution. The sample size was determined using Open Epi software based on data from a previous study on the Pediatric Balance Scale; the calculation involved a 95% confidence interval (two-sided), 5% alpha value, and 80% power [19].

The study was done after receiving the ethical approval of the Tehran University of Medical Sciences ethical committee with the reference number [NO: IR.TUMS.FNM.REC.1402.158] and registered in the Iranian registry of clinical trials under IRCT ID [IRCT20230903059340N1]. The first author is an international student at the Tehran University of Medical Sciences and conducted this study with the guidance of professors from various countries. Data collection took place in the researcher's home country (Iraq-Baghdad) under the supervision of Professor FMA at Sadr Al-Qanat Center and other Baghdad centers. All parents of the participants were made aware of the procedures that would be carried out in advance. The study only included children of the parents who signed a written consent form indicating their consent to participate.

**Figure 1:**
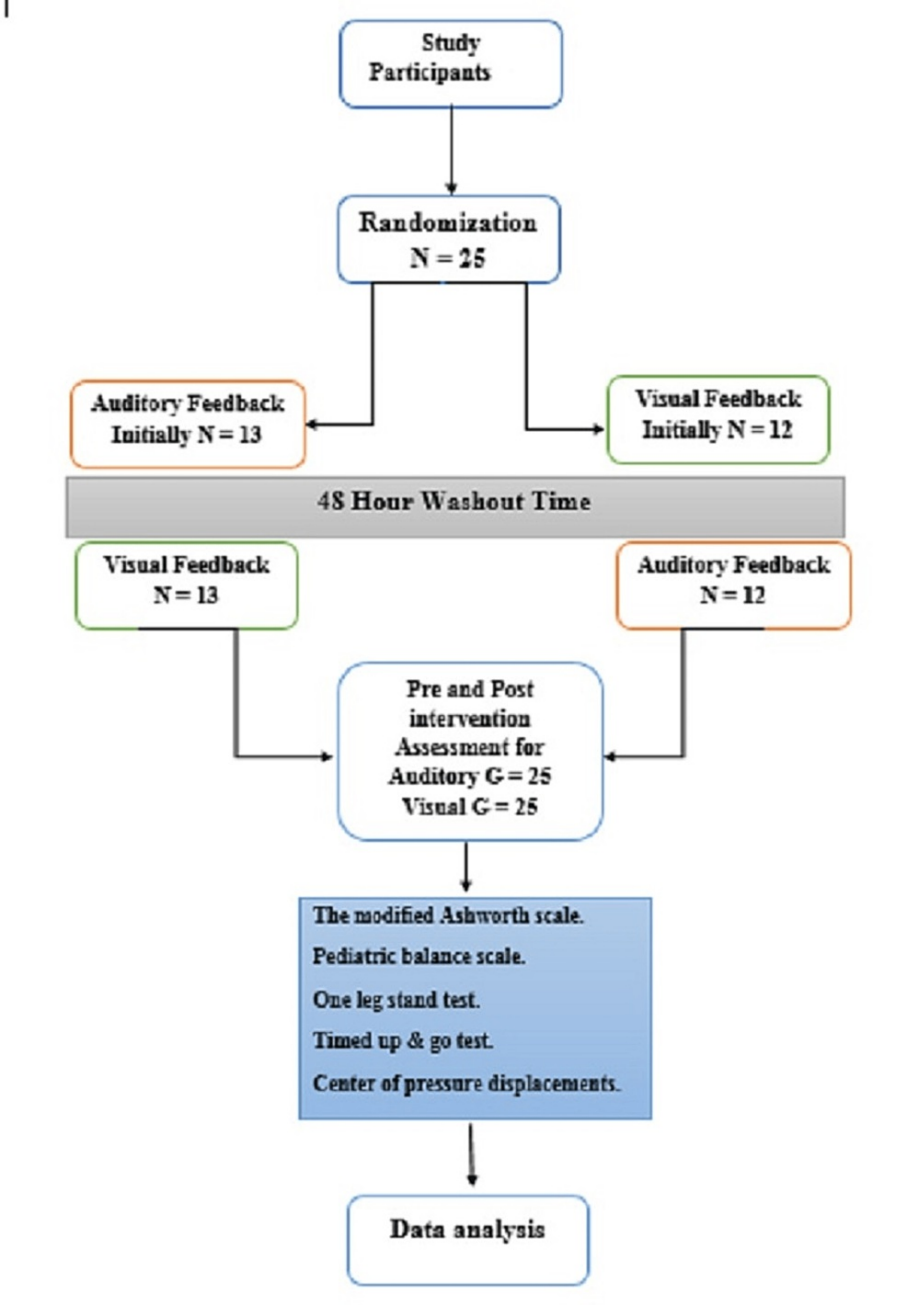
Flow diagram of participants in the study

The researcher employed a purposive sampling technique. The parents of patients meeting the criteria for the study were communicated by the researcher after being referred by the neurologist to participate in the research. Randomization was conducted utilizing a lottery-based approach to ascertain the order in which the groups would receive either visual or auditory feedback initially (Figure 1). The procedure, including the duration and content of the physiotherapy sessions, was explained to the parents. A physiotherapist conducted the baseline and post-treatment assessments without knowledge of the group assignments.

Assessment

Demographic details were collected for each patient, including age, weight, height, BMI, and gender. Data was collected both at baseline and after the completion of the session. The evaluation of outcomes included:

*The Modified Ashworth Scale (MAS)* involves the initial extension of the patient's limb from a position of maximum flexion to maximum extension or vice versa. The specific muscles targeted for assessment were the quadriceps and ankle plantar flexor muscles on the affected side. The degree of spasticity was determined based on the following scale (Table 1):

**Table 1:** The modified Ashworth scale degree [20]

Degree	Description
0	No increase in tone
1	Slight increase in tone resulting in a catch when the limb was moved in flexion or extension
1+	Slight increase in muscle tone with a catch followed by minimal resistance throughout the range of motion (ROM)
2	More pronounced increase in tone, but still allowing for relatively easy flexion of the limb.
3	Significant increase in tone, making passive movement difficult.
4	Limb rigid in either flexion or extension.

*The Pediatric Balance Scale (PBS)* is a modified version of the Berg Balance Scale that is used to assess functional balance skills in school-aged children. The scale consists of 14 items that are scored from 0 points (lowest function) to 4 points (highest function), with a maximum score of 56 points [21].

Item descriptions of the PBS are sitting to standing, standing to sitting, transfers, standing unsupported, sitting unsupported, standing with eyes closed, standing with feet together, standing with one foot in front, standing on one foot, turning 360 degrees, turning to look behind, retrieving objects from the floor, placing an alternate foot on a stool, and reaching forward with an outstretched arm.

*One Leg Stand Test* was measured by the timed one-leg stance test. The patient stood on the affected leg unassisted with eyes open, calculating time started when the opposite foot left the ground; time stopped immediately when the opposite foot touched the ground [22]. 

*The Timed Up and Go Test (TUG)* was conducted to evaluate walking speed, utilizing measuring tape, a chair with an armrest, tape to mark the ground, and a stopwatch. A three-meter walkway was measured using the measuring tape. A piece of tape (or cone) was placed on one end of the walkway, with the front legs serving as the starting point. To prepare for the test, the patient sat in the chair and leaned against the back of it. Upon saying "go," the patient rose from the chair, walked to the marker in front of them, turned around upon reaching the marker, and then sat back down. The time taken by the patient to complete the test was measured, with the stopwatch starting and stopping when the patient's bottom made contact with the chair. Assistive devices like walkers or crutches were permitted and consistently documented. Additionally, the patient was given one practice trial before the timed performance [23].

*Center of Pressure (CoP)* changes serve as an indirect indicator of postural instability, reflecting an individual's capacity to sustain equilibrium. Wii Balance Board (WBB) (Nintendo Company, model RVL-021, China) was used to detect changes in the body’s CoP. Equipped with Bluetooth technology, the board is outfitted with four pressure sensors that are utilized for determining the user's center of pressure. This point is identified as the intersection between an imaginary vertical line passing through the center of pressure and the surface of the Balance Board. Through Bluetooth connectivity, the board establishes a link with a laptop running the Brainblox software (2017, United States). Subsequently, the patient stands on the board barefoot, evenly distributing body weight across both legs to achieve optimal balance for a duration of one minute. Following this, the values and diagrams of the horizontal and vertical displacements of CoP are displayed on the laptop screen [24-32].

Intervention

The patients were given conventional balance therapy followed by either auditory feedback or visual feedback intervention. After a 48-hour washout period, they received conventional balance therapy again before undergoing the alternative intervention initially assigned. Throughout the duration of the study, the parents of the patients were instructed not to provide any other physiotherapy intervention to their children.

Conventional Balance Exercises

**Figure 2:**
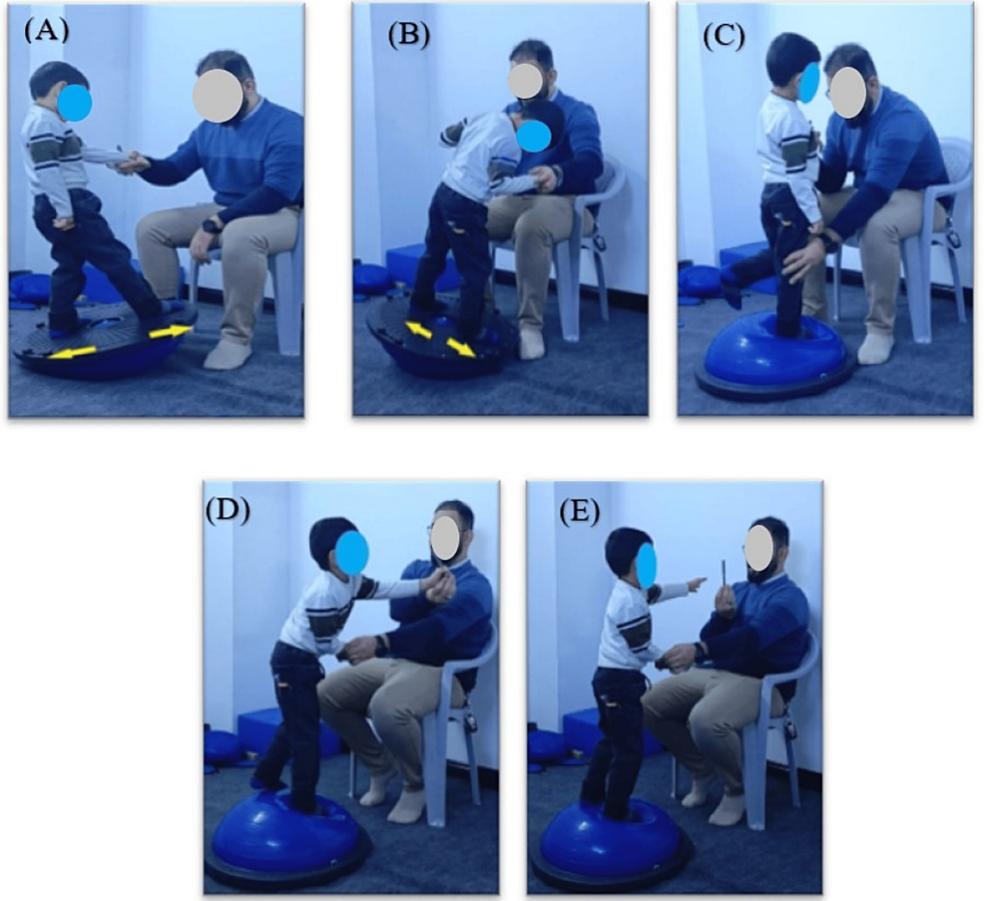
The conventional balance therapy exercises on a balance board: (A) the patient performed weight shifting from forward to backward exercise, (B) the patient performed weight shifting from side-to-side exercise, (C) the patient performed flamingo exercise, (D) oblique reaching exercise, and (E) forward reaching exercise

During the conventional balance exercises, the patients performed five exercises using the half-ball balance board (Buso) under the guidance and support of the physiotherapist, who provided that based on the patient's performance ability. These exercises included weight shifting from side to side, weight shifting from forward to backward, forward reaching, oblique reaching, and flamingo exercises (Figure 2). Each exercise consisted of 10-15 repetitions for 2-3 sets, lasting for six minutes. 

Visual Feedback Intervention

**Figure 3:**
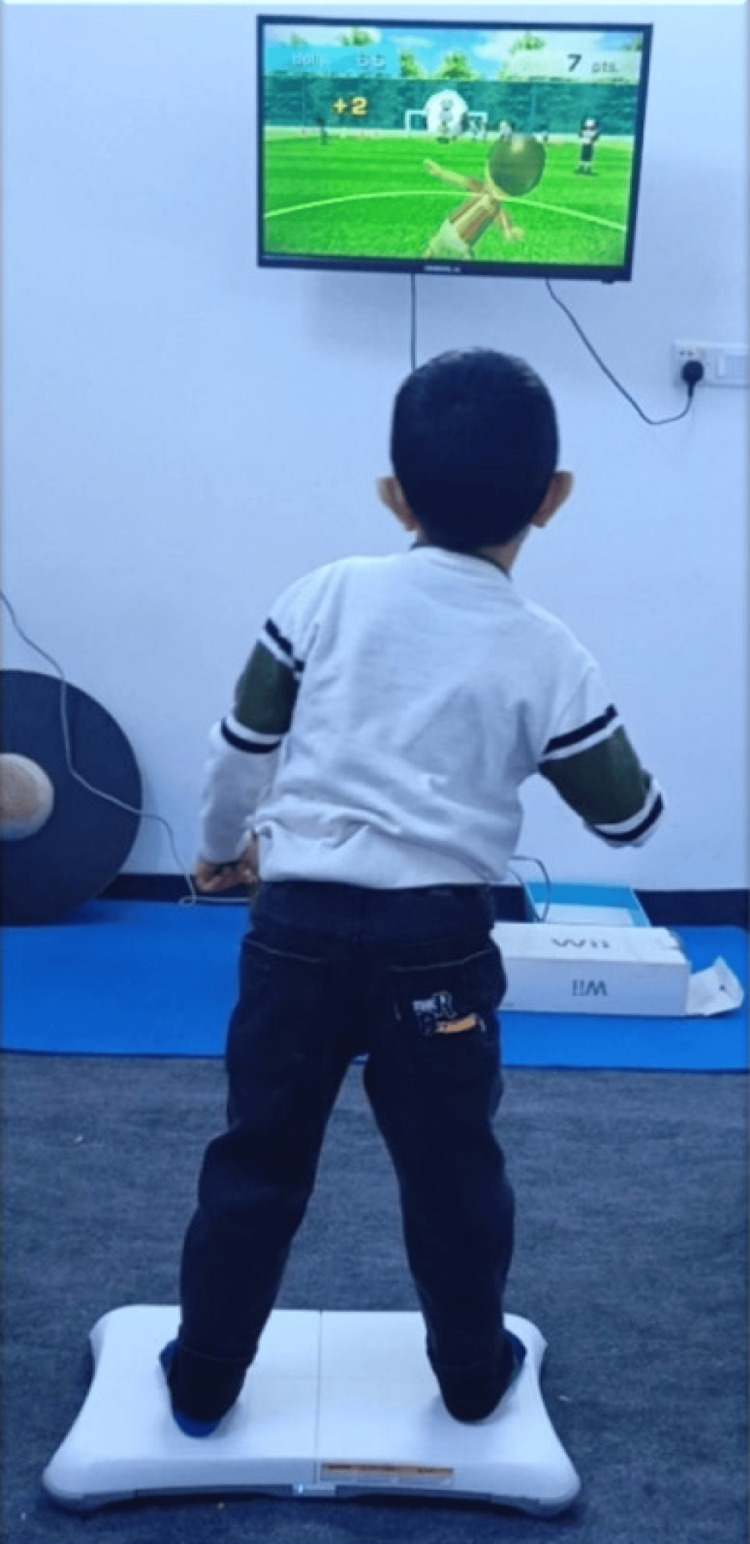
Visual feedback intervention: The patient did a soccer heading game by shifting his weight from side to side to hit the ball by his head

For visual feedback, a TV monitor was in front of the patient using a Nintendo Wii Fit gaming apparatus (model RVL-001, 2006 (EUR)). The patient was engaged in a selected balance game (soccer heading); the sound was muted, and the patient shifted his weight from side to side with the reflection of her/his body inside the game while he stood on the determined areas of the Wii balance board with opened eyes and the sound for 10 minutes in total (Figure 3).

The Auditory Feedback

**Figure 4:**
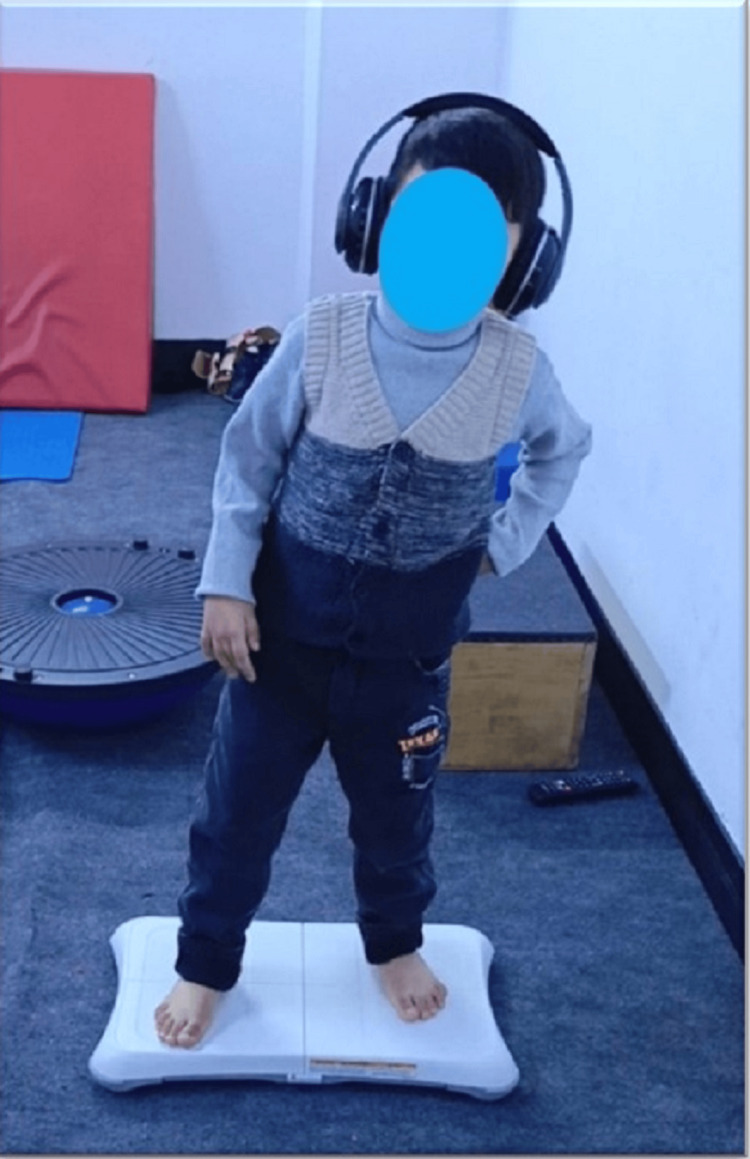
the patient is equipped with a wireless headset to receive auditory feedback intervention. This intervention involves providing the patient with instructions on how to perform the side-to-side exercise on the balance board.

The auditory feedback was presented through wireless stereo dynamic headphones version 5.0 connected to a mobile phone, which sends the voice via Bluetooth connection that the patient wore during the trial of doing the side-to-side weight shifting on the determined areas on the Wii balance board (Figure 4). The information was given as verbal instructions with low-volume background music on how to perform the exercise; the patient was instructed to look forward where there were no visual feedback effects in front; this interference lasted for 10 minutes in total.

Statistical analysis

To analyze the data, IBM Corp. Released 2019. IBM SPSS Statistics for Windows, Version 26.0. Armonk, NY: IBM Corp. was used. All data was tested by the Kolmogorov-Smirnov test to assess the normal distribution. To compare the demographic data, an independent t-test was used. To compare the effect of interventions on dependent variables within each group, the paired t-test (a parametric test) was employed to analyze the vertical displacement of the center of pressure variable in the visual feedback group. Conversely, the Wilcoxon Signed Rank Test (a non-parametric test) was utilized to analyze variables such as the modified Ashworth Scale, pediatric balance scale, one-leg stand, timed up and go, horizontal displacement of the center of pressure, and vertical displacement of the center of pressure in the auditory group. For comparing the mean differences between groups, the independent t-test (a parametric test) was conducted for the horizontal displacement of the center of pressure variable, while the Mann-Whitney U-Test (a non-parametric test) was used for all other variables. The level of significance was 0.05.

## Results

A total of 25 patients, comprised of 12 boys and 13 girls, participated in the present study, where each patient received the first intervention and after 48 hours of the washing period received the second intervention. The K-S test showed an abnormal distribution of the modified Ashworth scale (MAS), pediatric balance scale (PBS), one-leg stand, timed up-and-go (TUG), and vertical center of pressure (CoP) displacement (p-value < 0.05), while horizontal CoP displacement showed a normal distribution (p-value > 0.05).

The patients had an average age of (8.04) years with a standard deviation of (2.05), an average weight of (25.7) kg with a standard deviation of (8.97), an average height of (1.21) meters with a standard deviation of (0.15), and an average BMI of (20.7) kg/m2 with a standard deviation of (5.16).

Based on the findings obtained from the Wilcoxon Signed Rank Test, it was observed that both auditory and visual groups demonstrated equal significant differences in the MAS for quadriceps and ankle plantar flexor muscles (p-value = 0.03, p-value = 0.001, respectively), the one-leg-stand test (p-value = 0.00), and the TUG test (p-value = 0.00) before and after the intervention. Furthermore, the horizontal and vertical (CoP) of the visual feedback group demonstrated a significant difference (p-value = 0.001, p-value = 0.02). Conversely, there was no significant difference observed in the horizontal and vertical CoP of the auditory feedback group (p-value = 0.91, p-value = 0.71, respectively), and there was no significant difference observed in the PBS for both groups (p-value = 0.317) (Table 2).

**Table 2:** The comparison of the pre- and post-intervention values in the auditory feedback and visual feedback groups separately (N = 25 in each group) * Significant difference.

	Auditory group				Visual group			
Variables	Pre-intervention mean (SD)	Post-intervention mean (SD)	Mean Difference	P-value	Pre-intervention mean (SD)	Post-intervention mean (SD)	Mean Difference	P-value
Modified Ashworth grade of quadriceps muscles	1.32 (0.47)	1.00 (0.00)	-0.32	0.003*	1.32 (0.47)	1.00 (0.00)	-0.32	0.003*
Modified Ashworth grade of ankle plantar flexor muscles	1.4 (0.50)	1.00 (0.00)	-0.4	0.001*	1.4 (0.50)	1.00 (0.00)	-0.4	0.001*
Paediatric Balance Scale	51.84 (2.15)	52.00 (2.31)	0.16	.317	51.84 (2.15)	51.92 (2.12)	0.08	.317
Timed up & go test (second)	9.06 (2.55)	8.19 (2.29)	-0.87	.00*	8.87 (2.56)	7.46 (2.33)	-2.41	.00*
Time of one leg stand test (second)	1.47 (1.77)	1.79 (2.18)	0.32	.00*	1.29 (0.89)	9.70 (38.83)	8.41	.00*
Horizontal displacement of center of pressure (cm)	3.71 (2.38)	3.43 (2.18)	-0.28	.91	3.68 (2.52)	1.51 (1.21)	-2.17	.001*
Vertical displacement of center of pressure (cm)	2.21 (1.58)	2.65 (4.13)	0.44	.71	2.03 (1.59)	1.33 (0.96)	-0.7	.02*

The results of the Mann-Whitney U test revealed that there was a significant difference between the two groups regarding the TUG (p-value = 0.006) and one-leg stand (p-value = 0.017). Also, the independent sample T-test showed that there were significant differences between the two groups in regards to the mean horizontal CoP improvement (p-value = 0.03); however, there was no significant difference in MAS (p-value = 1.00), PBS (p-value = 0.98), or vertical CoP (p = 0.26) (Table 3).

**Table 3:** Comparison of the mean of variables between auditory feedback group and visual feedback group (n= 25 in each group) S: Significant difference, N.S: Non-significant, * Mann-Whitney U, ** T – test.

Variables	Mean Difference Within groups		Mean Difference between groups	P-value
	Auditory G	Visual G		
Modified Ashworth grade of quadriceps muscles	- 0.32	- 0.32	- 0.64	1.00 N.S
Modified Ashworth grade of ankle plantar flexor muscles	- 0.4	- 0.4	- 0.8	1.00* N.S
Paediatric Balance scale	0.16	0.08	0.24	.98* N.S
Timed up & go test (s)	- 0.87	- 2.41	- 3.28	.006* S
One-leg stand test (s)	0.32	8.41	8.73	.017* S
Vertical displacement of center of pressure (cm)	0.44	- 0.7	0.26	.26* N.S
Horizontal displacement of center of pressure (cm)	0.28	- 2.17	-1.89	.03** S

## Discussion

This study was conducted to compare the single-session effectiveness of two physical treatment interventions (auditory feedback versus visual feedback) in conjunction with conventional balance therapy in children with hemiplegic spastic cerebral palsy. The research findings indicate that incorporating both auditory and visual feedback, along with conventional balance therapy, during a single session can lead to significant improvements in the modified Ashworth scale (MAS), one-leg stand, and timed up-and-go (TUG). However, it was observed that the visual feedback had a greater impact on enhancing the horizontal and vertical displacement of the center of pressure (CoP), whereas no notable enhancements were observed in the pediatric balance scale (PBS).

Various studies have been carried out to investigate the impact of visual and auditory feedback on gait or walking in children with cerebral palsy. While some studies have focused on the effects of visual feedback or auditory feedback separately, there is a lack of research comparing the individual effects of auditory feedback and visual feedback on comprehensive balance outcome measures in hemiplegic spastic CP. These outcome measures include the MAS, PBS, one-leg stand, TUG, and displacement of the CoP.

The findings of our study align with previous research conducted by Seo et al. (2000), which concluded that both visual and auditory feedback were effective in enhancing standing balance in children with cerebral palsy [18]. Additionally, the results of the current study are consistent with the work of Dong-Hyun and Sung-Jin (2015), who found that both visual and auditory feedback groups exhibited greater improvement in CoP displacement compared to the control group in chronic stroke patients [19]. A study by Chang-Kyo and Yoo (2016) on the effects of balance training with visual feedback in children with spastic cerebral palsy demonstrated statistically significant differences in TUG and CoP, further supporting the results of the current study [33]. Moreover, research conducted by Annick et al. (2005) suggested that balance training with visual feedback could be beneficial in reducing the amplitude of CoP displacement during quiet standing in school-age children diagnosed with hemiplegic cerebral palsy [34].

In a systematic review conducted by Chen. et al. (2017), it was demonstrated that visual feedback proved to be an effective intervention for improving motor function in children with CP [35]. Furthermore, the findings indicated that a six-session Nintendo Wii balance board (NWBB) program led to a reduction in ankle spasticity in the plantar flexors and enhanced static standing balance in young individuals with spastic CP, as evidenced by the research of Gatica-Rojas et al. (2019) [36]. On the other hand, a study by Baram and Lenger (2012) demonstrated that patients with cerebral palsy-related gait disorders could benefit from training involving visual and auditory feedback cues [17]. Additionally, a study by Lee et al. (2015) suggested that sit-to-stand training with biofeedback might improve sit-to-stand performance and balance ability in stroke patients.

Furthermore, biofeedback is not only used for treating muscle weakness caused by fractures, ligament injuries, or nerve surgery but it is also employed in the rehabilitation of patients with upper motoneuron damage. This includes individuals who have suffered from strokes, traumatic brain injuries, or cerebral palsy, to facilitate neuromuscular reeducation. In addition to its role in transmitting feedforward commands from Purkinje cells, the motor cortex also functions as a feedback controller, receiving input from sensory and association cortices. As a result, the incorporation of biofeedback can have an impact on the motor command within the association cortex by utilizing auditory and visual signals. By tapping into previously unused neural circuitry, biofeedback has the potential to influence motor commands through the neural circuits present in the association cortex [37].

A biofeedback system developed for postural control aims to transmit information to the central nervous system regarding postural balance or orientation in order to enhance performance [38,39]. This is achieved through the utilization of somatosensory input enhancement, which improves balance control by providing immediate visual, auditory, electrotactile, and vibrotactile biofeedback based on detected performance data [40]. Levac D et al. (2012) recently conducted a scoping review and proposed several potential explanations for how virtual reality therapy can improve motor skills in children with cerebral palsy [41]. These explanations include the promotion of problem-solving skills and cognitive engagement during play from the user's perspective, as well as increased motivation and neuroplasticity changes. From the perspective of the virtual reality system or game properties, it enables the practice of repetitive task-oriented and task-specific activities in a virtual environment that closely resembles the real world. Furthermore, it allows for the adjustment of task difficulty, the provision of visual and/or auditory feedback, and the opportunity for interaction with others [35]. The visual feedback's characteristics may contribute to the greater effectiveness of visual feedback in improving the horizontal and vertical displacement of CoP compared to auditory feedback in the present study.

The findings indicated a notable disparity in the level of spasticity in the quadriceps and ankle plantar flexor muscles before and after the intervention in both groups. This outcome aligns with the research conducted by Gatica-Rojas et al. (2019), which explored the impact of Nintendo Wii balance board games (visual feedback) combined with an exercise program. The study demonstrated a decrease in ankle plantar flexor spasticity among children and adolescents with spastic cerebral palsy. It is highly probable that the significant changes in spasticity are also linked to the enhancements in balance following the intervention with the Nintendo Wii Balance Board (NWBB) [36]. Otherwise, the inclusion of weight shifts during training may have contributed to the strengthening of the relevant ankle muscles [34]. This not only helps to reduce non-functional muscle activations that can affect balance control but also improves overall muscle function. Another study conducted by Sharma et al. (2015) aimed to compare the effects of neuromuscular electrical stimulation with or without biofeedback in children with diplegic cerebral palsy. The findings of the study demonstrated that patients who underwent biofeedback and neuromuscular electrical stimulation experienced greater improvements compared to those who only received neuromuscular electrical stimulation and conventional exercises. Electrical stimulation has the potential to enhance the strength of antagonist muscles, thereby aiding in the alleviation of spasticity in the agonist muscle. The augmentation of motor unit recruitment or the elevation of firing frequencies through biofeedback appears to be the most effective approach in elucidating the observed increase in strength [42].

In a similar vein to the findings of the current study regarding the improvement in the TUG test and CoP, a study conducted by Yun. and Yoo et al. (2016) aimed to investigate the effects of balance training with visual feedback on children with spastic cerebral palsy who received physiotherapy sessions focusing on balance and walking using a three-dimensional balance trainer that incorporated visual feedback. The results indicated a significant increase in both the TUG test and CoP [33]. Likewise, another study conducted by Jae Ho Park and Chung et al. in 2016 explored the impact of visual feedback and auditory feedback on the balance and gait abilities of stroke patients. The robotic gait-training group in this study received general physical therapy along with robotic gait training. The intervention was found to be effective in improving the Berg balance scale and TUG test [43]. Furthermore, Seo et al. (2000) conducted a study to determine a more effective balance training method for children with cerebral palsy who were undergoing exercise therapy, and visual-auditory feedback training through a computer connected to a footplate was used to measure the lateral weight movement of the patients. The researchers concluded that visual and auditory feedback was effective in improving standing balance [18]. Additionally, Baram and Lenger (2012) conducted a study to examine the effects of gait training with visual and auditory feedback cues on the walking abilities of patients with cerebral palsy. Visual cues were generated by a virtual reality apparatus attached to the eyeglass frame, while auditory feedback cues were delivered through earphones in the form of a clicking sound in response to each step. The study demonstrated that training with visual and auditory feedback cues can lead to improvements in gait speed and length [17].

Despite the diminished differences observed in the results of PBS in this study, there was no statistically significant distinction. It is speculated that the duration of the intervention period was insufficient to yield a statistically significant influence on the pediatric balance scale.

Based on our current understanding, no previous research has incorporated the one-leg stand test, as utilized in this study. The results indicated that both interventions, in addition to conventional balance therapy, led to an increase in the duration of standing on the affected leg, a key indicator of balance capability. This enhancement could be attributed to the positive influence on dynamic and static balance abilities achieved through biofeedback as well as the strengthening of extensor muscles through balance exercises, as highlighted by Yun and Yoo et al. (2016). Their findings suggest that three-dimensional balance trainers may enhance dynamic and static balance by integrating various senses through visual feedback, strengthening extensor muscles through vertical movements focused on the knee joint, and promoting active center of gravity movements within the base of support. Furthermore, Sackley and Lincoln (1997) proposed that visual feedback training utilizing proprioceptive senses is effective in enhancing symmetrical posture and reducing sway. Essentially, the movements of a three-dimensional balance trainer engage visual, proprioceptive, and vestibular senses in conjunction with visual feedback, leading to heightened sensory integration and ultimately improved balance capabilities [33].

Recommendation

It would be beneficial to investigate the long-term effects of intervention for the treatment of children with CP, so further research is needed to determine the long-term effects as well as follow-up of these treatment approaches.

## Conclusions

The research findings suggest that utilizing both auditory feedback and visual feedback alongside conventional balance therapy can be beneficial in treating children with hemiplegic spastic cerebral palsy. This approach has shown effectiveness in improving outcomes measured by the MAS, one-leg stand, TUG, and horizontal CoP. Additionally, incorporating visual feedback may further enhance results, including improvements in vertical CoP.
